# Clinical Applications of Artificial Intelligence in Cardiovascular Imaging: Where Do We Stand?

**DOI:** 10.3390/life16030507

**Published:** 2026-03-19

**Authors:** Archit A. Singhal, Tiffany Bowyer-Howell, Nikant Sabharwal, Andrew Lewis, Andrew R. J. Mitchell, Oliver Rider, John A. Henry

**Affiliations:** 1 Oxford Heart Centre, John Radcliffe Hospital, Oxford University Hospitals NHS Foundation Trust, Oxford OX3 9DU, UK; archit.singhal@msd.ox.ac.uk (A.A.S.); nikant.sabharwal@ouh.nhs.uk (N.S.); andrew.lewis@cardiov.ox.ac.uk (A.L.); 2 Oxford Centre for Clinical Magnetic Resonance Research, University of Oxford, John Radcliffe Hospital, Oxford OX3 9DU, UK; oliver.rider@cardiov.ox.ac.uk; 3 University of Oxford Medical School, John Radcliffe Hospital, Oxford OX3 9DU, UK; tiffany.bowyer-howell@some.ox.ac.uk; 4 Department of Cardiology, Jersey General Hospital, Gloucester Street, St. Helier JE1 3QS, Jersey; an.mitchell@health.gov.je

**Keywords:** cardiovascular imaging, artificial intelligence, echocardiography, computerised tomography, nuclear cardiology, cardiac magnetic resonance imaging

## Abstract

Cardiovascular imaging is essential in the diagnosis, phenotyping and prognostic assessment of cardiovascular disease. However, longstanding limitations constrain the accuracy, throughput, and scalability of cardiovascular imaging techniques. Artificial intelligence (AI) has demonstrated a diverse range of potential benefits across modalities, including echocardiography, computerised tomography, nuclear imaging, and magnetic resonance imaging. These benefits include automated quantification of key heart parameters, ability to improve traditional disease detection and phenotyping, and image reconstruction. While the use of AI in clinical workflows is still largely emerging, its significance is becoming established through numerous promising studies. The evidence reviewed indicates that AI can meaningfully enhance disease management, clinical operations and patient experience when used alongside physician expertise. However, several challenges restrict the widespread clinical implementation of AI, including a lack of robust prospective evidence, regulatory hurdles, bias in training datasets, and ethical drawbacks such as data privacy and accountability. Future developments should prioritise large-scale prospective and multicentre validation and address practical and ethical barriers to ensure AI can be utilised safely and effectively in clinical settings. This narrative review comprehensively analyses advances in AI-driven cardiovascular imaging with a focus on clinical implementation.

## 1. Introduction

AI is rapidly transforming cardiovascular imaging. Using machine learning and deep learning methods, contemporary systems now assist across the workflow, from scan planning and acquisition to automated segmentation, quantification and structured reporting to disease detection and data-driven phenotyping [1]. These capabilities are enabling earlier recognition of pathology, uncovering image signatures beyond human perception (e.g., radiomics-derived textural features), and improving risk stratification (e.g., CT-based plaque analysis and CT-FFR) [1]. Crucially, AI is beginning to assist in phenotyping heterogeneous syndromes such as heart failure with preserved ejection fraction (HFpEF), supporting the discovery of subgroups that may benefit from targeted therapies [2]. Taken together, these advances position AI to help revolutionise cardiovascular imaging.

**Figure 1:**
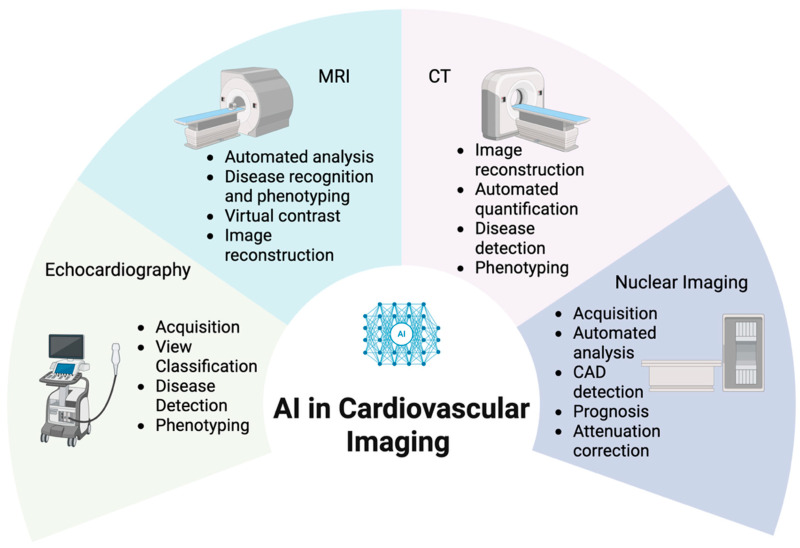
AI in cardiovascular imaging across modalities. Schematic overview of common AI applications in major cardiovascular imaging modalities. Abbreviations: AI, artificial intelligence; CAD, coronary artery disease; CT, computerised tomography; MRI, magnetic resonance imaging. Image created with BioRender.

There has been an explosion of research and early clinical deployment across echocardiography, cardiac computerised tomography (CT), nuclear cardiology, and cardiovascular magnetic resonance (CMR) imaging (Figure 1). Recent reviews comprehensively cover technical foundations of AI tools [1,3,4]. This narrative review aims to build upon this by summarising key developments modality by modality, emphasising clinical applications and potential future uses. We also discuss key practical and ethical considerations for safe and scalable implementation.

## 2. Artificial Intelligence in Echocardiography

### 2.1. Current Challenges in Echocardiography

Echocardiography remains the cornerstone of cardiac imaging due to its accessibility, portability, and diagnostic breadth, yet it faces well-recognised limitations. Image acquisition and interpretation are time-consuming, labour-intensive, and subject to inter-observer variability [5,6,7]. Manual contouring and measurement are operator-dependent and inconsistently reproducible, while retrospective measurement extraction is laborious and often incomplete, leading to significant under-utilisation of data [8]. Furthermore, differentiating cardiac pathologies, such as the aetiologies of left ventricular hypertrophy (LVH), including hypertrophic cardiomyopathy (HCM), hypertensive heart disease (HHD), and cardiac amyloidosis (CA), remains diagnostically challenging, especially in early disease stages when echocardiographic changes are subtle [9].

Artificial intelligence offers the potential to transform echocardiography by automating acquisition, quantification, and interpretation, thereby improving reproducibility, scalability, and clinical efficiency [10]. The integration of AI into echocardiographic practice has progressed rapidly over the past decade, encompassing tasks from view classification and chamber segmentation to disease detection, phenotyping, and prognostication.

### 2.2. Automated View Classification

A key barrier to applying AI in downstream echocardiographic tasks, such as automated quantification and disease detection, lies in the complexity and variability of imaging views. Unlike other imaging modalities, echocardiography involves multiple acoustic windows, often obtained in no fixed order; hence, a system must first recognise the view before analysis can proceed [11,12]. Developing a robust automated view classifier is therefore foundational for all advanced AI operations.

Naser et al. [11] addressed this challenge using 2D and 3D convolutional neural networks (CNNs) trained on real-world transthoracic echocardiograms (TTEs) and point-of-care ultrasound (POCUS) clips. Both networks achieved excellent performance on standard TTEs (2D CNN: accuracy 96.8%, area under the curve (AUC) 0.997; 3D CNN: accuracy 96.3%, AUC 0.998). Performance remained strong even on noisier POCUS data (2D CNN: accuracy 98.4%, AUC 0.999; 3D CNN: accuracy 95.0%, AUC 0.996). These results confirm that automated view classification is reliable even under suboptimal imaging conditions, extending potential utility to emergency and bedside settings. However, as with many AI-echocardiography models, the model in this work was trained and validated within a single centre, raising questions about generalisability across institutions, equipment vendors, and populations.

### 2.3. Automated Quantification: Ejection Fraction, Volumes, and Strain

Quantifying cardiac chamber size and function remains one of the most subjective components of echocardiography interpretation. Inter-observer variability in left ventricular ejection fraction (LVEF) or left atrial volume index (LAVi) can be significant, impacting patient management [8]. AI-based quantification seeks to standardise measurements, reduce noise, and accelerate analysis, providing reproducible results in seconds rather than minutes.

Early proof-of-concept work by Madani et al. [13] showed that deep learning (DL) algorithms could classify echocardiographic views and measure cardiac function with accuracy comparable to that of board-certified echocardiographers. Zhang et al. [14] extended this by demonstrating automated segmentation of cardiac chambers across five standard views, enabling accurate calculation of chamber volumes, mass, LVEF, and longitudinal strain.

More recently, Hu et al. [15] developed a fully automated DL pipeline encompassing data curation, measurement extraction, and rigorous quality control. In over 14,000 studies, automated measurements demonstrated low systematic bias compared with manual reference values (LVEF bias −1.8 percentage points; LAVi bias +3.3 mL/m^2^), albeit with relatively wide limits of agreement (±14.9 percentage points for LVEF and ±15.9 mL/m^2^ for LAVi). Variability increased in low-quality images but narrowed substantially in high-quality studies, and performance was consistent across major disease groups. While these results are comparable to reported inter-observer variability in routine echocardiography, the retrospective design, single-vendor data, and exclusion of lower-quality studies highlight the need for prospective, multicentre validation before clinical deployment.

In a prospective randomised trial an AI model for cardiac chamber annotation was non-inferior to expert sonographers, supporting the technical feasibility of automated quantification in a clinical setting [16]. Complementary work by Liu et al. [17] confirmed multicentre reliability of AI-derived LVEF, reinforcing the generalisability of such approaches beyond single-institution datasets. While these studies do not directly assess workflow efficiency or observer variability, they provide important evidence that AI-based quantification can achieve expert-level performance across diverse settings, forming a foundation for future evaluations of its impact on reproducibility, efficiency, and reporting standardisation.

### 2.4. Disease Detection and Phenotyping

#### 2.4.1. Heart Failure with Preserved Ejection Fraction

HFpEF remains a diagnostic challenge owing to its heterogeneity and lack of a single imaging or biochemical biomarker [18]. Many patients present with borderline or discordant findings, and scoring systems such as H2FPEF or HFA-PEFF have limitations [19]. AI can uncover subtle, multi-dimensional patterns in echocardiographic data, offering standardised diagnosis, phenotyping, and risk stratification.

In a landmark study, Akerman et al. [20] applied a 3D CNN to single apical-four-chamber clips to identify patients with clinically defined HFpEF. The model achieved 87.8% sensitivity and 81.9% specificity for HFpEF classification in independent multicentre testing. Importantly, the AI model reclassified many indeterminate cases from traditional scoring systems and carried prognostic value, with AI-positive patients exhibiting a 1.9-fold higher mortality during follow-up. Moreover, the study highlighted that many scans miss key imaging parameters used in conventional scoring systems, overcoming this problem with a single clip.

Beyond binary detection, machine learning (ML) enables phenotyping of HFpEF. Shah et al. [21] used unsupervised clustering of echocardiographic and clinical variables to reveal three phenotypes with distinct risk profiles and outcomes, findings replicated by Lancaster et al. [22] and Samad et al. [23], the latter integrating over 170,000 echocardiograms and electronic health record (HER) data to predict all-cause mortality (AUC 0.82). These AI-derived phenogroups outperform traditional clinical scores, supporting the feasibility of precision medicine in heart failure. Integrated multimodal approaches combining echocardiography with genomics, proteomics, and ECG data [21] may ultimately identify subgroups likely to benefit from targeted therapies.

#### 2.4.2. Hypertrophic Cardiomyopathy (HCM)

Detecting cardiomyopathies can be particularly challenging because of overlapping echocardiographic features. Echocardiography is often the first imaging tool for suspected HCM, but early disease can be visually subtle. A 2017 study demonstrated that a deep learning algorithm trained solely on 2D images could identify HCM with an AUC of 0.93 and cardiac amyloidosis with an AUC of 0.87, correlating moderately with LV mass (r = 0.23–0.36), indicating that AI captures latent image features beyond human recognition [14]. Later studies reported even higher performance (AUC ≈ 0.98) in distinguishing LV hypertrophy aetiology; however, this has yet to be tested in external validation cohorts [24].

Recent work using machine learning on routine echocardiography parameters achieved AUC values of 0.92–0.98 across four algorithms, with added strain data further improving accuracy [25]. Another model distinguished physiological (athletic) from pathological hypertrophy with 96% sensitivity when age was considered. Texture-based deep learning techniques can also delineate LVH aetiologies [9,26,27]. These tools promise earlier, scalable detection of HCM and its mimics, enabling timely and tailored management.

#### 2.4.3. Cardiac Amyloidosis

Cardiac amyloidosis diagnosis is often delayed because echocardiography findings overlap with hypertensive or hypertrophic cardiomyopathy. Given the emergence of disease-modifying therapies for transthyretin cardiac amyloidosis (ATTR-CA), early recognition is important. A multicentre deep learning model [28] trained on single apical-four-chamber clips achieved an AUC of 0.93, sensitivity of 85%, and specificity of 93%, outperforming conventional scores (AUC 0.73–0.80) and maintaining accuracy across amyloid subtypes. Other studies have confirmed high discrimination using both handcrafted and texture-based features [29,30]. A multimodal fusion model combining echocardiography and EHR data also achieved AUROC ≈ 0.94 [31], underscoring the value of multimodal integration. Clinically, such AI tools could enable automated screening of echocardiography databases, flagging high-risk patients for confirmatory scintigraphy or cardiac MRI. The FDA-cleared Us2.ai platform now includes an amyloidosis detection feature, representing translation from research to clinical practice [32].

#### 2.4.4. Coronary Artery Disease (CAD) and Ischaemia

AI has also shown promise in identifying regional wall motion abnormalities (RMWAs) on routine and bedside echocardiograms. Lin et al. demonstrated deep learning detection of RMWAs with AUC values of 0.90 (standard echocardiography) and 0.85 (bedside echocardiography) [33]. In stress echocardiography, the PROTEUS trial [34] found that AI assistance improved decision-making consistency among less experienced clinicians, bridging experience gaps. The ongoing EASE trial [35] is evaluating EchoGo Pro, an AI-driven stress echocardiography platform, for diagnostic accuracy and workflow efficiency. These developments point to AI’s potential to reduce interpretive variability and expedite non-invasive CAD assessment.

### 2.5. AI-Guided Acquisition and Probe Assistance

AI-driven acquisition guidance can democratise echocardiography, allowing novice operators to acquire diagnostic-quality images. In a small study, nurses with minimal echocardiography experience used a vendor-independent AI guidance system (trained on >5 million observations) to obtain interpretable images for LV size/function in 98.8% and right ventricle (RV) size in 92.5% of patients [36]. Similar results were achieved by medical students using the same system [37]. Such guidance tools reduce operator dependence, improve reproducibility, and expand echocardiography access in emergency care, primary care, and low-resource settings.

### 2.6. Monitoring Disease Progression and Treatment Response

AI’s role extends beyond diagnosis to longitudinal monitoring. In cardio-oncology, automated analysis of serial echocardiograms can identify subclinical declines in LVEF, allowing early intervention before symptomatic heart failure develops [38]. One study has shown that handheld AI-enabled ultrasound systems can detect LVEF < 50% earlier than traditional workflows [39]. Similarly, machine learning models can classify aortic stenosis severity and predict timing of valve replacement more accurately than conventional metrics, reducing unnecessary follow-up scans and optimising surveillance strategies [39,40].

AI is redefining echocardiography from a manual, operator-dependent modality into an automated, data-driven discipline. Early applications, from view classification and quantification to disease detection and phenotyping, have already achieved expert-level accuracy. Emerging use cases in acquisition guidance, risk prediction, and longitudinal monitoring further illustrate its transformative potential. To ensure safe and equitable adoption, future work must focus on multicentre validation, ethical governance, and integration into clinical workflows. With continued progress, AI-enabled echocardiography is poised to deliver faster, fairer, and more precise cardiovascular care.

Table 1 summarises key trials in the clinical application of AI in echocardiography.

## 3. Artificial Intelligence in Computerised Tomography

### 3.1. Current Challenges of CT

Computerised tomography, in particular, coronary computerised tomography angiography (CCTA), is often the front-line imaging technique used to assess suspected CAD [4]. This is due to its relatively quick scan time, non-invasive nature, and the accuracy of images produced. However, limitations remain, including radiation exposure [41], commonly found artefacts due to stents or calcification, and difficulties obtaining functional information. Recent AI implementations in CT have shown potential to overcome such limitations, assisting with image reconstruction, faster analysis [42], and automation of quantitative values [43]. Such developments facilitate earlier diagnosis and prognostication of cardiovascular disease [42], which may reduce the occurrences of myocardial infarction and improve morbidity and mortality [44].

### 3.2. Image Reconstruction

CCTA has a high diagnostic accuracy in detecting obstructive coronary stenosis [45]. However, images produced from this technique are susceptible to degeneration, most significantly at lower radiation doses [42]. Previously, CCTA has involved a compromise between radiation dose and image quality, with improvements in one aspect resulting in a detriment to the other; lowering the current of the X-ray tube results in insufficient photons in the projection area and, thus, elevated quantum noise [46]. However, with implementation of reconstructive AI algorithms, scans produced from low-dose radiation can be restored to produce a clearer image with reduced noise and removed artefacts [47]. A deep learning reconstruction (DLR) model, trained on paired samples with and without noise, was trialled by Tatsugami et al. to reduce noise and improve image quality in CT scans. The model significantly reduced image noise and enhanced image quality compared to the hybrid iterative reconstruction (IR) algorithm [48]. Otgonbaatar et al. supported this conclusion whilst also quantitatively measuring the quality at which stents were imaged, which also showed improved resolution [49]. De Santis et al. found similar conclusions in addition to high levels of correlation between Adaptive Statistical Iterative Reconstruction-Veo (ASiR-V) and DLR in the diagnosis of CAD [50]. TrueFidelity is the first FDA-approved DL image reconstruction technique. Benz et al. demonstrated that this programme allows for a 43% reduction in radiation dose from that used in CCTA whilst limiting image noise and retaining integrity of stenosis severity and plaque composition and volume [51]. Clinically, improved image quality with noise reduction may reduce re-scanning and improve the diagnostic value of CT scans, whilst reducing radiation exposure.

### 3.3. Coronary Artery Calcium

Coronary artery calcium (CAC) is a helpful marker of subclinical atherosclerosis and is beneficial for cardiovascular risk stratification [52], with the 2021 ESC guidelines naming CAC scoring as the best-established imaging modality for improving assigned risk strata [53]. CAC scoring from CT helps prioritise patients for preventative therapies and regular surveillance, whilst also improving adherence to statin therapy and lifestyle improvements [54,55].

Traditional Agatston scoring, however, requires dedicated ECG-gated non-contrast CT, manual or semi-automated segmentation, and expert review [56]: all of which limit large-scale implementation of CAC quantification in clinical settings. Agatston 2.0 has recently been introduced, incorporating AI-based approaches to improve detection of small, semi-calcified plaques that may previously have gone undetected [57]. Despite this, many limitations surrounding acquisition and workflow remain. Further AI developments, therefore, offer a compelling solution enabling rapid, reproducible CAC detection and quantification from routine CT data [58]. Zeleznik et al. showed strong agreement between a deep learning model and manual scoring in over 5000 individuals and test–retest reliability (Spearman’s correlation 0.92), illustrating the efficacy of their model [59]. A DL model that can score CAC from both gated coronary CT and non-gated chest CT has been developed [55]. The model showed excellent agreement with the gold standard of CAC quantification (a gated CT exam) and discernment between coronary and non-coronary calcification that may otherwise produce false positive results.

The ability for AI to support “opportunistic screening” in relation to assessing CAC from non-gated chest CTs offers major workflow and patient-care benefits, avoiding additional radiation exposure and scan time. Additionally, 2016 guidelines from the Society of Cardiovascular Computerised Tomography and the Society of Thoracic Radiology have recommended the implementation of CAC reporting on all non-gated chest CTs due to the high prognostic value of this measurement and the benefit of reducing extra scan requirements, especially when 6.6 of the 7 million US patients determined eligible for lung scanning were also deemed to expect benefit from CAC screening [60]. Recent review articles [56,58] highlight the promise of AI-enabled CAC scoring in improving workflow efficiency, inter-observer reproducibility, and population-level risk detection. Remaining challenges include variation in acquisition parameters (gated vs. non-gated scans and the use of contrast), differing reconstruction kernels, and potential overestimation in the presence of motion or stents. This emphasises the need for multicentre validation and regulatory standardisation if routine implementation is to be reached. Nevertheless, automated CAC quantification represents one of the most mature AI applications in cardiac CT due to measurable time savings, excellent agreement with manual scoring, and tangible potential to expand early cardiovascular risk assessment.

### 3.4. Disease Detection and Phenotyping

Implementation of AI in CT is progressing beyond simply improving image quality and quantifying data. It can extract multi-dimensional signals indicative of disease. Such markers include plaque burden and composition [43], functional ischaemia, perivascular inflammation, and adipose tissue phenotyping. Such tools allow conversion of morphological CT data to prognostic biomarkers, which can better stratify risk and personalise cardiac management.

#### 3.4.1. Plaque Quantification and Phenotype

Deep learning pipelines now enable automated, volumetric quantification of coronary plaque with detail about the proportion of that which is calcified, non-calcified, and low attenuation from CCTA [61]. Recent large multicentre studies, such as REVEALPLAQUE [62], show that DL-derived plaque information correlates closely with expert manual measures and provides independent prognostic information for myocardial infarction and major adverse cardiac events. The Scottish Computerised Tomography of the HEART (SCOT-HEART) trial found low-attenuation plaque burden to be the strongest predictor of myocardial infarction in those presenting with stable chest pain [63]. These automated measures facilitate reproducible tracking of plaque progression and allow plaque phenotype to be incorporated into risk models. Commercial AI platforms such as Cleerly provide automated, standardised quantification and phenotyping of coronary plaque burden from CCTA, as well as assessment of stenosis severity relevant to downstream ischaemic risk, enabling consistent plaque phenotyping across clinical workflows [64].

#### 3.4.2. Stenosis Assessment and Functional Ischaemia

Machine learning (ML) methods have been used to predict lesion-specific ischaemia from CCTA-derived fractional flow reserve (CT-FFR) [65]. ML-based CT-FFR methods produce estimates that compare favourably with invasive FFR whilst reducing unnecessary invasive angiography and diagnosis time. Emerging studies and reviews suggest that CT-FFR shortens processing times in comparison with computational fluid dynamics approaches while retaining clinically acceptable diagnostic accuracy [66,67,68]. CT-FFR has not yet been validated in various patient demographics, including those with coronary stents and bypass grafts [69].

#### 3.4.3. Epicardial Adipose Tissue (EAT) and Systemic Phenotypes

Automated DL segmentation of epicardial adipose tissue (EAT) is now feasible from CCTA [70], producing rapid measures of EAT volume and attenuation that are associated with numerous cardiovascular diseases, including CAD and atrial fibrillation [71]. Large-scale and multicentre DL tools for EAT quantification have been developed and externally validated, enabling integration of adipose phenotypes into risk stratification, potentially identifying high-risk subjects. West et al. [70] developed a programme to quantify EAT with efficacy in patients with technical challenges such as difficult anatomy and scan artefacts.

#### 3.4.4. Integrated Prognostic Models

Beyond single biomarkers, AI enables multimodal prognostic models that fuse CCTA-derived features such as plaque burden and perivascular inflammation with clinical data to produce individualised risk predictions [72]. Several studies show that these AI-derived models outperform conventional scores and can reclassify risk to guide preventive therapy [73]. This technology has been shown to accurately predict cardiovascular events and mortality in patients with non-obstructive coronary disease, the population in which approximately one third of events occur [73,74]. Moreover, this AI-Risk algorithm has been shown to prospectively reclassify risk categories compared to traditional risk classifications, such as QRISK3 and SCORE [74,75], and to change clinical management in a significant proportion of patients [74,75]. In this study, coronary inflammation assessment was performed using CaRi-Heart^®^ v2.5, developed by Caristo Diagnostics, which quantifies the perivascular fat attenuation index (FAI) in each coronary artery and integrates age- and sex-adjusted FAI percentiles with plaque burden and clinical variables to derive an AI-Risk score [75]. This commercially available platform shows how AI-based multimodal prognostic models are being translated into routine CCTA workflows.

In this context, the Oxford Risk Factors and Non-invasive imaging (ORFAN) study comprised over 40,000 patients undergoing clinically indicated CCTA across multiple centres, with longitudinal follow-up for major adverse cardiac events (MACE) [74]. Analysis demonstrated that increased perivascular FAI in each coronary artery was associated with an increased risk of cardiac mortality and MACE, with additive prognostic value across vessels. Crucially, the predictive value of coronary FAI was independent of traditional cardiovascular risk factors, in addition to the presence and extent of CAD. These findings supplement emerging evidence that FAI can enhance prognosis beyond anatomic indicators [76]. This study indicated that an AI-assisted risk prediction tool integrating FAI, atherosclerotic plaque burden, and patient risk profiles can provide meaningful risk reclassification in those undergoing routine CCTA. This approach may facilitate more targeted implementation of preventative strategies, such as anti-inflammatory therapies; however, future studies should aim to establish whether AI-guided risk stratification and treatment improve clinical outcomes.

**Figure 2:**
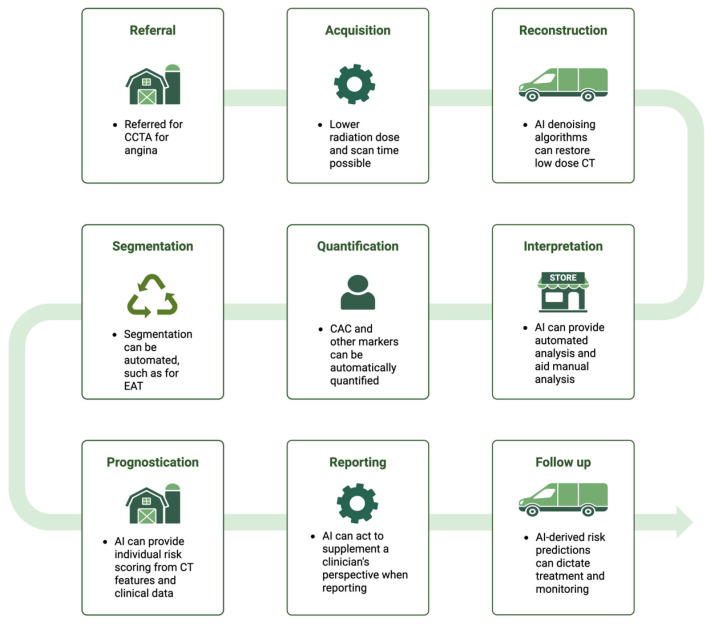
Clinical workflow example of AI-enabled cardiovascular imaging from referral to decision-making. Illustrative pathway showing where artificial intelligence (AI) can support the continuum of care in cardiovascular CT imaging, from referral for coronary CT angiography (CCTA) through image acquisition and reconstruction (including low-dose protocols and AI-based denoising), automated segmentation and quantification of imaging biomarkers (e.g., coronary artery calcium and epicardial adipose tissue), and AI-assisted interpretation. AI-derived outputs may inform prognostication and structured reporting, ultimately supporting downstream clinical decisions regarding treatment, follow-up, and monitoring. Abbreviations: AI, artificial intelligence; CAC, coronary artery calcium; CT, computerised tomography; CCTA, coronary computerised tomography angiography; CV, cardiovascular; EAT, epicardial adipose tissue. Image created with BioRender.com.

Table 2 summarises key trials in the clinical application of AI in CT. Figure 2 shows an illustrative example of clinical workflow of AI-enabled cardiovascular imaging from referral to decision making.

## 4. Artificial Intelligence in Nuclear Cardiology

### 4.1. Current Challenges in Nuclear Cardiology

Single-photon emission computerised tomography (SPECT) and positron emission tomography (PET) are methods of myocardial perfusion imaging (MPI). These are primary tests for diagnosing CAD and risk stratification of such patients [77], but they face familiar constraints seen with other modalities: there is a trade-off between radiation dose and scan time versus image quality [78], imaging artefacts and noise degrade specificity, interpretation quality is variable across centres/readers [79], and a range of limitations surround quantitative PET blood-flow tools [80]. Quantification of myocardial blood flow shows an opportunity to elevate nuclear cardiology to increase reproducibility and prognostic ability, yet such programmes are operationally complex and not universally available [81]. In response to such restraints, the European Association of Nuclear Medicine, as of 2022, has set expectations for responsible AI adoption, with optimism that this can improve the quality of medical practice and compensate for a radiologist shortage [82]. Potential risks of such implementation have, however, been acknowledged, with an emphasis on the need for constant revalidation of AI tools, transparent reporting, and multicentre and multigroup validation of trials.

### 4.2. CAD Detection from SPECT MPI

DL models trained on large, multicentre SPECT polar maps have been indicated to outperform conventional quantitative metrics, such as total perfusion deficit (TPD), for identifying obstructive CAD [83]. Early multicentre work showed improved per-patient and per-vessel detection compared with TPD. This work has been extended to create a model able to automatically combine upright and supine MPI polar maps to predict obstructive CAD more efficaciously than TPD (disproving the previously thought single-view limitation) [84]. Importantly for clinical practice, explainable DL overlays and vessel-specific probabilities supplement professional interpretation (accuracy and calibration) when used as decision support, rather than replacing readers, despite high standalone diagnostic accuracy [85]. This was demonstrated prospectively with blinded readers, where a significantly higher rate of correct diagnosis of CAD was seen with the addition of DL results as compared with interpretation using solely clinical history, stress, and quantitative perfusion. Most recently, DL predictive outputs have been translated back into quantitative perfusion scoring, producing AI-enhanced perfusion scores [86]. This method indicated greater predictive ability for obstructive CAD than traditional analysis or the DL model in isolation. Furthermore, this approach presents physicians with AI-enhanced data in a familiar format, potentially making deployment into clinical practice easier. Clinically, these studies demonstrate the ability of AI to improve and harmonise diagnostic performance across readers and labs, especially where interpretive variability and attenuation artefacts limit specificity.

### 4.3. Prognosis and Management Decisions from SPECT

Beyond disease diagnosis, explainable DL approaches applied to SPECT MPI could directly predict major adverse cardiac events, adding incremental prognostic information beyond visual/quantitative analysis and clinical covariates [87]. Previous ML algorithms have been able to predict such events; however, they have disadvantages that the DL model can overcome, including the input of many variables (70 in one model, with half requiring manual collection) and the requirement for software-dependent quantification, which could reduce generalisability of such models. The model produced by Singh et al. was trained and tested in large, multi-site cohorts (e.g., REFINE SPECT) with external validation, and provides explanations of the image regions contributing to the prediction of adverse events to guide clinical reasoning. Hu et al. demonstrated the ability of ML to automate cancellation of rest SPECT scans following stress SPECT scanning [88]. This ML approach has been indicated as having significantly higher prognostic safety compared with that achieved by physician interpretation of stress images or application of clinical selection rules. Whilst SPECT MPI traditionally includes both rest and stress scans, many sources indicate the redundancy of rest scans when a normal stress scan has been produced. Stress-only imaging can reduce patient radiation dose by 60% [89] whilst reducing cost and increasing throughput from a clinical perspective. Arsanjani et al. demonstrated that an ML model combining clinical and quantitative features from SPECT scans can predict revascularisation need in patients with suspected CAD, with a level of accuracy at least equal to that of expert readers [90]. This may aid in determining which patients would benefit from invasive coronary angiography, as opposed to those needing only medical therapy without the need for additional downstream testing. This reduces time spent on interpretation by professionals and prevents patients from undergoing unnecessary invasive procedures whilst ensuring those most in need receive such treatments. Overall, AI has the potential to derive comprehensive risk scores that can aid in physician interpretation and determination of patient management. It could additionally facilitate automated protocol decisions, which can streamline care whilst maintaining quality of patient care.

### 4.4. PET MBF/MFR: Automated Analysis and Phenotyping

Conventional relative MPI, particularly gated SPECT, can underestimate the extent of ischaemia in multivessel and left main coronary artery disease because perfusion is assessed relative to the “best” myocardial segment rather than in absolute terms [91]. Absolute myocardial blood flow (MBF) and myocardial flow reserve (MFR) measured with PET therefore add important diagnostic and prognostic value by unmasking diffuse and balanced ischaemia and characterising microvascular dysfunction. Reflecting this, absolute MBF quantification has been assigned a Category I add-on for a PET MPI study by the Centres for Medicare and Medicaid Services [92]. Despite operational challenges, quantification of MBF supports improved diagnosis of CAD, risk stratification, and patient selection for coronary interventions and medical therapy.

DL models trained on PET polar maps have demonstrated the ability to identify impaired MFR and classify cardiovascular risk traits, including sex and smoking, from perfusion patterns throughout the left ventricle [93]. This can contribute to an automated triage system and the structured reporting of quantitative flow, allowing for prioritisation of high-risk patients. Evidence indicates that AI can lower the barrier to routine MBF/MFR use by automating segmentation/analysis and highlighting high-risk physiology that might be missed by relative perfusion alone. Routine implementation of MBF/MFR has a range of clinical outcomes in diagnosis/prognosis, but the supplementation of such quantification by AI can help streamline processing and save time in composing reports for physicians.

### 4.5. FDG PET in Inflammatory Cardiomyopathies

FDG PET with myocardial glucose suppression is central to diagnosing cardiac sarcoidosis, but current workflows require manual reorientation to perfusion datasets and myocardial segmentation, which are labour-intensive and variable. A recent study developed a 3D U-Net DL algorithm for automated myocardial segmentation of inflammatory FDG PET, trained on 316 cardiac sarcoidosis studies with left ventricular contours derived from perfusion images and tested in a 50-patient subset [94]. Compared with the standard software segmentation, the DL approach improved clinical readability in over 90% of cases and achieved performance comparable to that of a trained technologist, while substantially reducing left ventricular displacement and angulation errors.

### 4.6. Dose/Time Reduction and Image Quality (SPECT and PET)

A practical route to increasing accessibility of nuclear MPI is AI-enabled denoising to recover diagnostic quality from low-count acquisitions. A 2021 study by Aghakhan Olia et al. assessed a DL algorithm that was able to effectively denoise and recover underlying information from half- and quarter-dose SPECT images [95]. However, the number of clinically acceptable reconstructed images at a quarter dose of radiation was reduced to 80%, with many of the nuclear scans being those from patients with moderate to severe risk conditions. This once again emphasises the constant compromise in MPI and CT between image acquisition and radiation dose. But promisingly, the ability to obtain diagnostically sufficient images from half-dose scans using AI shows the ability of such algorithms to progress towards a standard of low radiation exposure but high image quality. Similar findings were established by Sun et al. [96] and Du et al. [97] with the use of algorithms to denoise SPECT images.

For ^82^Rb cardiac PET, self-supervised DL has been developed to simultaneously denoise dynamic frames and correct positron range, potentially improving visual quality and quantitative robustness [98].

Notoriously, acquisition time, patient motion, or tracer supply constrain image quality. Development of DL denoising models can enable shorter scans or lower radiation doses without sacrificing interpretability, benefiting patients, physicians, and healthcare systems.

### 4.7. Attenuation Correction When CT Is Not Available

SPECT frequently utilises CT-based attenuation correction (AC) to improve diagnostic accuracy. DL has the ability to produce synthetic AC SPECT images suitable for clinical interpretation [99]. This is particularly promising for community labs without hybrid SPECT/CT. Shanbhag et al. [99] developed a conditional GAN-based model, DeepAC, of which the predictive performance of the resulting images was significantly improved. DeepAC has the promising ability to correct attenuation artefacts without obscuring true defects through overcorrection. Yang et al. [100] deduced similar outcomes from their DL model, FA-ACNet. CT-free AC allows the benefit of repeat-scan reduction due to artefact limitation to be expanded to hospitals lacking hybrid SPECT/CT scanners. This allows further integration of AI with the prospect of reducing radiation dosage and reducing scan time/costs by providing physicians with images holding greater diagnostic abilities.

### 4.8. Amyloidosis Imaging

Diagnosis of ATTR-CA, an increasingly established cause of heart failure in older patients, by visual interpretation of SPECT (the clinical gold standard of interpretation) is inherently subjective, particularly in cases of lower uptake [101]. Technetium-99 whole-body scintigraphy is almost pathognomonic of ATTR CA except for rare false positives, commonly in relation to light-chain amyloidosis [102]. Manual quantification of SPECT myocardial ^99m^Tc-pyrophosphate imaging increases reproducibility of scan interpretation in relation to ATTR CA but is not clinically used due to being so labour-intensive. DL methods may enable fully automated, volumetric quantification of ^99m^Tc-pyrophosphate uptake by segmentation of coregistered CT attenuation maps. Miller et al. [101] produced a model with potential to support physician interpretation or risk determination by associating quantified ^99m^Tc-pyrophosphate uptake with cardiovascular mortality or hospitalisation due to heart failure.

Furthermore, ML models have shown success in automating CA diagnosis; a recent multicentre study reported the first successful automated CA diagnosis using a SPECT/CT hybrid radiomics model [103]. This model integrates numerous ML diagnostic/prognostic algorithms to achieve increased diagnostic accuracy as compared with the use of single markers. This model outperformed those using traditional metrics, as the utilisation of multiple factors for assessment increases specificity of diagnosis and allows this model to classify amyloid subtype (ATTR vs. AL) with important consequences for treatment.

With careful governance and multicentre validation in concordance with ASNC/EANM guidance, AI can make nuclear imaging more accurate, consistent, streamlined, and accessible. This has the potential to improve patient outcomes whilst reducing radiation exposure and the requirement for additional invasive procedures. In clinical settings, AI highlights its potential in reducing scan/interpretation time and improving diagnostic and prognostic quality of nuclear imaging, increasing the value of this modality.

Table 3 summarises key trials in the clinical application of AI in nuclear imaging.

## 5. Artificial Intelligence in Cardiac Magnetic Resonance Imaging

Cardiovascular magnetic resonance is uniquely positioned to deliver comprehensive, radiation-free assessment of cardiac structure, function, perfusion, and tissue characterisation. However, the modality has long been limited by labour-intensive analysis and relatively long, motion-sensitive acquisitions. Over the past few years, AI, and especially deep learning, has begun to reshape nearly every step of the CMR workflow, from acquisition and reconstruction to automated reporting and disease-specific interpretation. Recent reviews concur that AI’s most immediate value is pragmatic (speed, standardisation, and robustness), while early disease-detection tools and synthetic-contrast techniques hint at new diagnostic capabilities that could broaden access to CMR and improve clinical yield [104,105].

### 5.1. Automated Cine Analysis and Reporting

Automated analysis of cine CMR is the best-established AI application in routine practice. Fully convolutional networks trained on large annotated cohorts (e.g., UK Biobank) can delineate endocardial/epicardial borders across the cardiac cycle in seconds and derive left and right ventricular volumes, mass, and ejection fractions with human-level accuracy [106]. In a 4875-subject study, mean absolute differences between automated and manual measurements were only 6.1 mL (LVEDV), 5.3 mL (LVESV), and 8.5 mL (RVEDV), with segmentation Dice scores up to 0.94, well within expert inter-observer variability. These systems remove the tedious manual contouring step while standardising how key metrics are measured [106].

Clinical deployment has followed. Arterys Cardio DL received FDA clearance in 2017 for automated, editable ventricular segmentation in CMR, an early proof that AI-derived function metrics can meet regulatory standards. Such platforms generate results rapidly and enable consistent, reproducible reports that fit seamlessly into PACS workflows [107].

### 5.2. Disease-Specific Recognition on Routine CMR

AI’s pattern-recognition strength is increasingly used to identify cardiomyopathies on cine imaging and late gadolinium enhancement (LGE) datasets. For cardiac amyloidosis, a CNN trained on multi-view LGE images achieved excellent diagnostic performance (AUC ≈ 0.98), matching a handcrafted-feature ML model that emulated expert reading, supporting AI as a triage “scout” that surfaces a diagnosis for confirmatory evaluation [108]. In arrhythmogenic cardiomyopathy, AI methods that quantify RV motion/strain from cine series have shown feasibility for objective, reproducible assessment of wall motion abnormalities central to Task Force Criteria and may assist in classification among suspected cases [109,110].

Radiomics and deep learning are also being studied to differentiate overlapping phenotypes (e.g., ischaemic vs. dilated cardiomyopathy) from cine features, a task that can be challenging in daily practice [111]. Radiomics in CMR means turning routine images (cine, LGE, and mapping) into dozens-to-hundreds of numeric “texture” and shape features that capture subtle patterns the eye may miss. In practice, this can help with diagnosis and risk prediction while keeping workflows simple because it uses scans clinicians already acquire [112]. Reviews show growing evidence across cardiomyopathies, though standardisation and external validation remain important for clinical rollout [112,113]. In HCM, cine radiomics can triage who is likely to have LGE-detectable fibrosis (potentially reducing the need for contrast use), and multicentre work combining cine radiomics with deep learning has identified HCM patients without scar on LGE, useful as a screening aid [114,115]. Beyond detection, LGE radiomics (capturing scar texture/shape rather than just percent LGE) adds prognostic signal for outcomes including sudden cardiac death, complementing conventional markers [116,117]. Outside HCM, radiomics has been tested in amyloidosis for both diagnosis and prognosis using LGE and T1-based features, supporting its role as decision support alongside expert reading [118,119].

A related theme is phenotyping cardiomyopathy with reduced systolic function. Early studies using non-contrast cine radiomics (and ML) show feasibility for distinguishing ischaemic from dilated cardiomyopathy and even detecting infarct-related scar without gadolinium, extending earlier cine radiomics work on myocardial infarction [111,120].

Overall, radiomics offers a low-friction way to extract more value from CMR, flagging fibrosis in HCM, enriching risk models, and helping separate ischaemic from dilated phenotypes, provided that models are built and validated with robust, standardised pipelines before routine use [113]. These tools are not yet a replacement for clinician interpretation, but they can serve as decision support, highlighting patterns or regions of concern, reducing oversight risk, and promoting systematic, standardised reporting across readers and centres [105].

### 5.3. Synthetic Contrast (Virtual Native Enhancement)

Perhaps the most provocative advance is “virtual contrast” imaging. LGE remains the reference for focal scar/fibrosis, yet it adds time, cost, and IV contrast exposure. In 2021, investigators introduced Virtual Native Enhancement (VNE), which uses deep learning to combine native T1 maps and cine images to synthesise LGE-like images without contrast [121]. In HCM, the study reported that VNE produced scar images of equal or better quality than conventional LGE, raising the possibility of gadolinium-free scar assessment [121]. Furthermore, Zhang et al. corroborated this, showing that VNE can produce better image qualities and was strongly correlated with LGE in quantifying scar size post MI [122]. These advances have the potential for shorter, cheaper, needle-free CMR exams, and safer imaging for patients with advanced kidney disease. Validation beyond HCM and ischaemic cardiomyopathy is ongoing, but early clinical research studies are already beginning to use the technology when conventional LGE is not possible [123].

### 5.4. Scar Quantification and Risk Stratification

For cardiomyopathies where scar burden influences management, AI delivers rapid, reproducible quantification. In HCM, an interpretable CNN segmented LV borders and scar on LGE, producing scar-percentage measurements that tightly correlated with expert analysis (r = 0.92) [124]. Such tools can standardise scar quantification for risk stratification and follow-up, reducing inter-observer variability that complicates decision-making (e.g., considering ICD placement in high-scar phenotypes) [124]. Similar fully automated frameworks are emerging for ischaemic scar and microvascular obstruction, poised to support post-MI risk stratification and therapy planning once validated prospectively [125].

### 5.5. Acquisition and Reconstruction: Faster, Freer-Breathing, More Robust CMR

Deep learning reconstructions now allow under-sampling whilst still delivering diagnostic cine images. In practice, this means shorter exams and fewer (or no) breath-holds, important for patients with dyspnoea, heart failure, or arrhythmias. Prospective comparisons show that free-breathing DL cine achieves non-inferior image quality and accurate LV volumes versus standard breath-hold cine; in some subgroups with poor breath-holding or arrhythmia, the DL approach performs as well or better for diagnostic quality [126,127,128]. Clinically, highly accelerated cine protocols have cut total cine time by ~66–80% (including single-heartbeat-per-slice options) without loss of quality, improving patient tolerance and freeing scanner time for added sequences or higher throughput [126,127,128].

On the back end, newer self-supervised and model-based (unrolled) reconstruction strategies reduce reliance on fully sampled training data and generalise better across vendors and protocols, a practical requirement for multi-site deployment. The upshot is more consistent image quality even when local protocols differ, which helps standardise reporting and longitudinal follow-up [129,130]. In 4D flow MRI, DL denoising and super-resolution shorten acquisitions and recover hemodynamic metrics that are otherwise limited by low resolution/noise, supporting more reliable assessment of flow patterns in the great vessels and, increasingly, the chambers [131,132]. AI also assists with motion/artefact detection and correction, which improves reader confidence and stabilises downstream quantification, particularly valuable in real-world scans where motion is common [133].

### 5.6. Tissue Characterisation and Quantitative Perfusion

For mapping, AI can standardise segmentation and reduce breath-holds. MyoMapNet-style networks estimate T1 from just a few images, enabling four-heartbeat T1 mapping with accuracy comparable to conventional techniques, useful for patients who struggle with long breath-holds and for busy lists needing predictable timing [134].

For stress perfusion, integrated pipelines now perform inline, fully automated pixel-wise MBF mapping on the scanner, auto-segment the myocardium, and output AHA-segment reports without user interaction. Clinically, this converts a complex, semi-quantitative exam into a standardised, objective test that is easier to compare across time and sites and less dependent on operator expertise [135,136]. Repeatability data in suspected CAD and multicentre feasibility studies support real-world use [137]. In disease-specific cohorts (e.g., HCM), inline perfusion mapping has already offered mechanistic insights into microvascular dysfunction with immediate potential to refine management [138].

Table 4 summarises key trials in the clinical application of AI in CMR.

## 6. Practical Considerations

Despite impressive technical advances, few AI tools in cardiovascular imaging have achieved widespread clinical use. Key barriers arise from inherited biases introduced during model development: homogeneous training cohorts that are not representative of real-world case mix, limited external validation, noisy or inaccurate annotations and outcome labels, and missing data [139]. Indeed, Wilkinson et al. have demonstrated that only 24% of published AI algorithms have been tested on external cohorts [140]. Underrepresentation of women, minorities and even low-risk populations in training datasets will cause inaccurate generalisation of model performance [141,142,143,144].

In addition, many AI studies in cardiovascular imaging report excellent discrimination (with AUC frequently > 0.90), yet high performance in development datasets does not necessarily translate into real-world effectiveness. Apparent performance may be inflated by spectrum bias, whereby models are trained on highly selected case–control cohorts with clear disease phenotypes rather than the heterogeneous and multimorbid populations encountered in clinical practice. Artificial or imbalanced disease prevalence within development datasets may further distort performance metrics and limit transportability. Moreover, reliance on internal validation alone, or on expert annotation as a reference standard rather than independent gold-standard confirmation, may overestimate true diagnostic accuracy.

It is also important to distinguish discrimination from calibration. While discrimination metrics such as AUC reflect a model’s ability to separate disease from non-disease, they do not assess whether predicted probabilities correspond to observed event rates. Poor calibration may substantially limit clinical utility, even when discrimination appears strong, particularly in risk prediction or triage settings.

Label noise, arising from imperfect annotations or diagnostic uncertainty, may also influence performance and generalisability. Such inaccuracies in ground-truth labelling can propagate systematic bias during training and contribute to performance degradation when models are deployed in new environments.

Moreover, much of the literature still emphasises algorithmic performance over evidence of patient benefit and cost-effectiveness; prospective and real-world evaluations remain comparatively sparse and often methodologically limited, underscoring the need for pragmatic trials and post-deployment monitoring [145]. For image reconstruction in particular, evaluations often lean on technical image-quality metrics or internal datasets rather than prospective, reader- and outcome-based studies, making it hard to guarantee diagnostic equivalence in day-to-day practice [146]. Future work should therefore prioritise prospective, multicentre validation, transparent reporting of reference standards, calibration analysis, and continuous real-world performance auditing following deployment.

Beyond technical performance, real-world implementation requires consideration of infrastructure, regulation, and workflow integration. Governance and privacy rules complicate data access and sharing, while integration into RIS/PACS and on-scanner workflows is non-trivial and must avoid placing additional burden on clinicians [147]. Regulatory pathways also differ internationally, with FDA clearance and CE marking reflecting distinct evidentiary thresholds. Reimbursement frameworks further influence adoption, as clinical utility alone does not guarantee financial sustainability.

Importantly, models validated on static datasets may experience dataset shift over time due to evolving imaging protocols, scanner upgrades, or demographic changes, potentially leading to performance degradation. Also, human–AI interaction introduces new failure modes, including automation bias and over-reliance on algorithmic outputs. Ongoing monitoring, transparent reporting, and governance frameworks are therefore essential for safe deployment.

A further practical barrier relates to the accessibility of appropriate engineering expertise for clinicians seeking to develop or implement AI tools. Effective translation typically requires collaboration between cardiovascular specialists, data scientists, software engineers, and regulatory experts within established academic or industry frameworks. Laboratories working in this space should demonstrate access to diverse and well-annotated datasets, experience in external validation and prospective study design, familiarity with regulatory and data-governance requirements, and the capability to integrate tools within existing clinical infrastructure.

Finally, formal health economic evaluation, including cost-effectiveness, return on investment, and system-level impact, will be essential to determine the sustainability of large-scale AI implementation in cardiovascular imaging.

## 7. Ethics

The ethical concerns surrounding the use of AI in cardiac imaging represent a significant barrier to its widespread clinical implementation. Core ethical challenges include preservation of patient confidentiality, accountability for decision-making, and mitigation of algorithmic bias.

Effective deployment of AI models requires the exchange of patient data and computational frameworks between numerous institutions and, potentially, international boundaries [148]. Such data sharing may conflict with patient privacy regulations and could compromise the integrity of informed consent if the patient is not explicitly advised on how their data may be used in both current and future applications [149]. In particular, the secondary use of routinely acquired imaging data for algorithm development raises important questions regarding data ownership, transparency, and the scope of consent, especially where datasets are repurposed for applications beyond the original clinical indication. Robust governance frameworks, de-identification standards, and clear communication with patients are therefore essential.

Algorithmic bias represents another major ethical concern. Models trained on demographically homogeneous datasets may inadvertently perpetuate or amplify existing healthcare disparities if performance differs across sex, ethnicity, socioeconomic status, or comorbidity profiles. Strategies to mitigate such bias include ensuring diverse and representative training cohorts, transparent reporting of subgroup performance, and ongoing post-deployment auditing to detect performance drift over time.

Furthermore, the use of AI models in healthcare raises queries in both the ethical and legal contemplation of accountability, particularly with the progressive development of autonomous models [4]. When model-derived outputs contribute to diagnostic or therapeutic decisions with potentially serious outcomes, the allocation of liability is often unclear; the extent to which the AI developers, clinicians, and institutions are responsible is controversial [150]. Until robust regulatory and ethical frameworks are established, AI should serve as support for decision-making, with the final interpretation and patient management resting with the clinician [4].

## 8. Future Work

Looking ahead, future work should aim to reflect the realities of clinical deployment. Methodologically, that means prospective, multicentre reader studies with external validation; transparent reporting using TRIPOD-AI for prediction models, CLAIM-2024 for imaging AI, and DECIDE-AI for early live testing; and endpoints that matter clinically (diagnostic yield, time-to-treatment, downstream management, throughput and costs) [151,152,153]. Technically, priorities include training for generalisability (vendor/protocol-agnostic strategies and harmonisation), privacy-preserving collaboration at scale (federated learning), and modern pretraining (self-supervised/foundation models) to reduce annotation burden and improve robustness [154] and improve reliability and reproducibility. This is particularly important for radiomics, where features and even segmentations can vary with acquisition and software, and standardised pipelines such as the Image Biomarker Standardisation Initiative (IBSI) need to be adopted [155]. Safe, sustainable operations also require calibrated uncertainty and continuous post-market performance monitoring, supported by emerging regulatory tools, such as the FDA’s Predetermined Change Control Plans and the EU AI Act’s high-risk framework [156,157]. Coupled with “last-mile” workflow design (seamless PACS/RIS integration and clear, structured outputs), these steps can turn AI from promising prototypes into reliable service tools that shorten and simplify exams, standardise quantification, and scale access, while keeping clinicians in charge.

## 9. Conclusions

AI is beginning to reform cardiovascular imaging and influence clinical practice. Across echocardiography, CT, nuclear imaging, and CMR, AI shows potential to aid in more consistent image acquisition, automated reporting, disease detection, phenotyping, and risk stratification. These advances could help to overcome persistent barriers to high-quality imaging, including operator dependence, variable interpretation, and lack of time for expert analysis. AI models show potential to improve consistency of quantified measurements, including LVEF, CAC scores, myocardial perfusion indices, and CMR-derived functional measures. Beyond this, AI may streamline workflows by reducing laborious reporting, shortening scan times, lowering repeat-scan rates, and detecting and phenotyping diseases earlier. This may improve clinical decision-making, whilst improving patient safety and expanding access to high-quality imaging, particularly in busy or resource-limited clinical settings. However, key limitations currently restrict the widespread integration of these models into clinical practice. These include the biased nature of datasets, lack of multicentre prospective validation, and unresolved ethical and practical challenges. Despite this, with robust validation and ethical integration, AI shows promise in strengthening every stage of cardiovascular healthcare as a decision-support tool to augment but not replace clinical expertise. Future research should aim to optimise convergence of the principal modalities and focus on evidencing improvements in patient outcomes rather than direct algorithmic performance.

## Figures and Tables

**Table 1:** Summary of representative studies evaluating artificial intelligence (AI) methods in echocardiography across: (i) view classification, (ii) automated quantification of cardiac chambers and function, (iii) disease detection/phenotyping, (iv) AI-guided acquisition, and (v) monitoring disease progression/treatment response. Columns summarise the study focus, citation, brief study design/approach, key outcomes, and main limitations as reported by the authors. Abbreviations: 2D, two-dimensional; 3D, three-dimensional; AI, artificial intelligence; AS, aortic stenosis; AUC, area under the receiver operating characteristic curve; AVR, aortic valve replacement; CA, cardiac amyloidosis; CAD, coronary artery disease; CE, Conformité Européenne (European Conformity) marking; CMR, cardiac magnetic resonance; CNN, convolutional neural network; DL, deep learning; EASE and PROTEUS, study/trial names; EHR, electronic health record; FDA, (U.S.) Food and Drug Administration; HFpEF, heart failure with preserved ejection fraction; HCM, hypertrophic cardiomyopathy; LAVI, left atrial volume index; LSTM, long short-term memory (network); LV, left ventricle/left ventricular; LVEF, left ventricular ejection fraction; LVH, left ventricular hypertrophy; MI, myocardial infarction; ML, machine learning; POCUS, point-of-care ultrasound; QC, quality control; RCT, randomised controlled trial; RMWA, regional wall motion abnormality; RV, right ventricle/right ventricular; TTE, transthoracic echocardiography; “vs.”, versus; “≈”, approximately.

Section	Condition/Focus	Study (ref)	Study Summary	Key Performance/Outcomes	Main Limitations
**View classification**					
	TTE & POCUS view recognition	**Naser 2024 [11]**	Retrospective single-centre study using 2D and 3D CNNs to classify views from TTE and POCUS clips.	Accuracy ≈ 95–98%; AUC ≈ 0.996–0.999 for TTE and POCUS.	Single-centre, vendor-homogeneous; external generalisability uncertain.
	Contrast & non-contrast echo views	**Zhu 2022 [12]**	Retrospective study using DL to classify contrast and non-contrast echo views.	Demonstrated robust automatic view classification across contrast and non-contrast studies.	Mainly development data; limited multi-vendor, multicentre validation.
	Early deep learning view classification	**Madani 2018 [13]**	Retrospective proof-of-concept DL model for view classification and function estimation.	View/function performance comparable to board-certified echocardiographers.	Early single-centre work; limited real-world and multicentre validation described.
**Automated quantification**					
	Multi-view chamber segmentation & function	**Zhang 2018 [14]**	Retrospective clinical dataset with DL segmentation of chambers in 5 standard views to compute volumes, LV mass, LVEF and strain.	Demonstrated fully automated chamber quantification from routine echoes.	No prospective or broad multi-vendor external validation reported.
	Large-scale automated pipeline (LVEF, LAVI)	**Hu 2025 [15]**	Large retrospective single-vendor pipeline for automated measurement extraction and QC across >14,000 studies.	Small bias vs. manual for LVEF and LAVI; performance consistent across disease groups.	Retrospective; vendor-homogeneous; low-quality studies excluded.
	AI vs. sonographers—prospective trial	**He 2023 [16]**	Prospective blinded randomised non-inferiority clinical trial comparing AI-based chamber annotation/function with expert sonographers.	AI cardiac function assessment non-inferior to sonographers. Time saving.	Conducted in controlled trial setting; long-term workflow and outcome effects unclear.
	Multicentre LVEF assessment	**Liu 2021 [17]**	Retrospective multicentre study of DL-based 2D echo LVEF assessment.	Reliable AI LVEF across centres; reduced inter-observer variability.	Focused on LVEF only; performance in poor image quality/complex pathology less clear.
**Disease detection—HFpEF**					
	HFpEF detection from single apical-4-chamber clip	**Akerman 2023 [20]**	Observational study using a 3D CNN on single apical-4-chamber clips to detect HFpEF.	Sensitivity 87.8%, specificity 81.9%; reclassified indeterminate scores; AI-positive patients had ≈1.9× higher mortality.	Single-view model in specific cohorts; external multicentre validation needed.
	HFpEF phenotyping via clustering	**Shah 2015 [21]**	Prospective cohort study phenomapping using unsupervised clustering of echo and clinical variables in HFpEF.	Three phenotypes identified with distinct risk profiles and outcomes, supporting precision medicine.	Pre-DL era; depends on chosen variables and cohorts.
	Diastolic function phenogroups	**Lancaster 2019 [22]**	Retrospective phenotypic clustering of LV diastolic function parameters.	Defined diastolic phenogroups with prognostic relevance.	Variable selection and retrospective design may limit generalisability.
	Echo + EHR for survival prediction	**Samad 2019 [23]**	Large retrospective ML study (>170,000 echoes + EHR) predicting survival from echo and clinical data.	AUC ≈ 0.82 for all-cause mortality; scalable prognostic modelling.	Observational “black-box” model; prospective impact on management not shown.
**Disease detection—HCM/LVH**					
	LVH identification on echo	**Yu 2022 [24]**	Retrospective DL model for LVH aetiology classification.	Reported AUC ≈ 0.98 for differentiating LVH causes.	No robust external validation cohorts; risk of overfitting.
	HCM diagnosis from routine echo (with strain)	**Farahani 2024 [25]**	Retrospective multi-algorithm ML using routine echo (including strain) to detect HCM and athletic vs. pathological LVH.	AUC 0.92–0.98; 96% sensitivity distinguishing athletic vs. pathological hypertrophy with age included.	Prospective validation and real-world deployment data limited.
	Texture/hybrid models for LVH aetiology	**Yu 2021 [26]** **, Wu 2023 [27]**	Retrospective texture-based and hybrid CNN–LSTM/ML models to separate LVH aetiologies (HCM, amyloidosis, other LVH).	Good discrimination of LVH causes from myocardial texture/sequence data.	Centre- and vendor-specific pipelines; little prospective outcome evidence.
**Disease detection—Cardiac amyloidosis**					
	Single-clip CA screening (multicentre)	**Slivnick 2025 [28]**	Multicentre DL model using a single apical-4-chamber clip for CA screening.	AUC 0.93; sensitivity 85%, specificity 93%; better than conventional scores.	Screening tool; requires confirmatory scintigraphy/CMR; implementation and cost-effectiveness pending.
	CA detection from routine echo measurements	**Chang 2024 [29]**	Retrospective ML using routine quantifiable echo measurements to detect CA.	AUC 0.84, sensitivity 0.82.	Dependent on complete, accurate measurements; not directly image-based. Single centre.
	Deep learning TTE for CA diagnosis	**Zhang 2023 [30]**	Retrospective DL-assisted TTE approach to diagnose CA.	Demonstrated that DL-assisted TTE can support CA diagnosis.	Single-centre; effect on diagnostic pathways and outcomes not reported.
	Commercial CA detection platform	**Us2.ai (FDA/CE) [32]**	Regulatory-cleared AI echo platform including an amyloidosis detection module.	FDA- and CE-cleared automated CA screening within routine echo workflows.	Proprietary algorithms; limited peer-reviewed performance data; real-world equity and adoption questions remain.
**Disease detection—CAD/ischaemia**					
	RMWA detection & function in MI	**Lin 2022 [33]**	Retrospective DL analysis of standard and bedside echoes in MI for RMWA and function.	AUC 0.91 (standard) and 0.85 (bedside) for RMWA; automated function quantification.	Focused on MI; broader CAD/pathology applicability not yet established. Retrospective design.
	Stress echo decision support (PROTEUS)	**PROTEUS trial [34]**	Stress-echo RCT comparing standard versus AI-augmented decision-making for coronary angiography.	AI was not inferior to standard decision-making. AI may support improved decision-making in less experienced clinicians.	Primarily improved reader consistency; limited hard clinical outcome data.
	AI-driven stress echo workflow (EASE)	**Mahdavi 2024—EASE [35]**	Mixed-methods real-world evaluation of EchoGo Pro AI stress echo platform.	Ongoing trial.	Ongoing trial.
**AI-guided acquisition**					
	Novice nurses guided by AI	**Narang 2021 [36]**	Prospective multicentre study of nurses with minimal echo experience using vendor-independent AI guidance for TTE acquisition.	Diagnostic LV size/function in 98.8% and RV size in 92.5% of patients.	Limited to specific views; small cohort; not a replacement for full sonographer studies.
	Medical students guided by AI	**Schneider 2021 [37]**	Prospective study of ultrasound-naïve medical students using ML probe guidance and AI LVEF estimation.	Novices obtained diagnostic loops; AI LVEF estimates agreed with reference.	Restricted protocols; long-term skill retention and impact on service delivery unknown.
**Monitoring disease progression/treatment response**					
	Aortic stenosis phenotyping and progression	**Sengupta 2021 [39]**	Retrospective ML framework using echo parameters to phenotype AS severity.	Identified distinct AS phenotypes and improved risk stratification, informing timing of valve intervention.	Observational; not yet linked to prospective management changes or outcome improvements.
	ML to optimise AS follow-up	**Sánchez-Puente 2023 [40]**	Retrospective ML models predicting AS progression and timing of valve replacement.	Improved AS risk stratification and prediction of AVR vs. conventional metrics; potential to streamline follow-up.	Requires prospective validation and health-economic evaluation across diverse populations and systems.

**Table 2:** Summary of key studies applying artificial intelligence (AI) in cardiac computerised tomography (CT), grouped by application: (1) image reconstruction; (2) coronary artery calcium (CAC) quantification; (3) plaque phenotyping; (4) adipose phenotyping; and (5) integrated risk modelling. Columns summarise the study focus, citation, brief study design/approach, key outcomes, and main limitations as reported by the authors. Abbreviations: AI, artificial intelligence; ASiR-V, Adaptive Statistical Iterative Reconstruction–V (iterative reconstruction algorithm); CAC, coronary artery calcium; CAD, coronary artery disease; CaRi-Heart, Cardiac Risk in the Heart (platform/study name); CCTA, coronary computerised tomography angiography; CT, computerised tomography; DL, deep learning; DLR, deep learning reconstruction; EAT, epicardial adipose tissue; FAI, fat attenuation index; FBP, filtered back projection; hybrid IR, hybrid iterative reconstruction; ICC, intraclass correlation coefficient; IR, iterative reconstruction; IVUS, intravascular ultrasound; MACE, major adverse cardiovascular events; MI, myocardial infarction; ORFAN, study name; QRISK3, cardiovascular risk prediction algorithm (version 3); ρ, Spearman’s rank correlation coefficient; SCORE, Systematic COronary Risk Evaluation risk score; SCOT-HEART, Scottish Computerised Tomography of the HEART trial; “≈”, approximately; “>”, greater than.

Section	Condition/Focus	Study (ref)	Study Summary	Key Performance/Outcomes	Main Limitations
**Image Reconstruction**					
	Noise reduction and image quality	**Tatsugami et al., 2019 [47]**	Retrospective study; DLR trained to suppress noise; CT from each patient reconstructed with hybrid IR and DLR	Significant reduction in image noise and superior image quality compared with hybrid iterative reconstruction	Small population; CT attenuation profiles only of selected areas; no confirmation of diagnostic accuracy
	Improved image quality and visibility of features	**Otgonbaatar et al., 2022 [49]**	Retrospective CCTA datasets (15/51 had stents); DLR, hybrid IR, FBP applied and reviewed	Improved noise reduction and enhanced spatial resolution of stents	Small population, did not evaluate blooming artefacts in stents
	CAD diagnosis from CCTA	**De Santis et al., 2023 [50]**	Prospective study; comparison of DLR with hybrid IR and FBP reconstruction	High correlation between DLR and ASiR-V in CAD diagnosis; DLR highest image quality	Vendor-specific implementation; broader validation required; no comparison with invasive coronary catheterisation; small, single-centre cohort
	Radiation dose reduction	**Benz et al., 2022 [51]**	Prospective study; patients underwent sequential normal and lower dose CT scans reconstructed with ASiR-V and DLR respectively	DLR enabled 43% radiation dose reduction; no significant impact on noise; stenosis severity and plaque characteristics preserved	No external reference standard
**Coronary Artery Calcium Quantification**					
	Automated CAC scoring	**Zeleznik et al., 2021 [59]**	Retrospective analysis of >20,000 individuals, prospective follow-up for cardiovascular events and death; DL model for automated CAC quantification from CT	Robust risk stratification; strong agreement with manual scoring; high test–retest reliability (ρ ≈ 0.92, ICC 0.993)	Lack of robust clinical evidence; retrospective design
	CAC scoring from gated & non-gated CT	**Eng et al., 2021 [55]**	DL-based CAC scoring of gated and non-gated CT; end-to-end models trained on robust reference standards	Excellent agreement of non-gated CAC with gated CT with reduced analysis time, good diagnostic performance, and reduced false positives	Retrospective design; gated and non-gated CTs performed at different times (<1 year)
**Plaque Phenotyping**					
	Automated plaque analysis	**REVEALPLAQUE, Narula et al., 2024 [62]**	Large multicentre study using DL-derived plaque metrics from CCTA	DL plaque measures correlated with IVUS segmentation	Lack of clinical outcomes; limitations of IVUS; no specific sub-analysis of each scanner
	MI risk prediction from plaque quantification	**SCOT-HEART trial, Williams et al., 2020 [63]**	Prospective trial using CCTA plaque analysis to predict MI	Low-attenuation plaque strongest predictor of myocardial infarction in stable chest pain	Not exclusively AI-driven; single technique used to analyse plaque; not all risk parameters accounted for
**Adipose Phenotyping**					
	Epicardial adipose tissue (EAT) quantification	**West et al., 2023 [70]**	DL segmentation of EAT from CCTA across multicentre datasets	Rapid, reproducible EAT quantification and prognostic value demonstrated	Lack of data limits conclusions about causes of noncardiac mortality and analysis of cardiac mortality
**Integrated Risk Modelling**					
	Individualised cardiovascular risk prediction from CCTA	**Oikonomou et al., 2021 [73]**	CaRi-Heart CCTA-derived FAI mapping with traditional risk factors to detect coronary artery inflammation	Improved prognostic performance over traditional risk factors alone, with over 30% reclassified	Clinical interpretation of FAI depends on many technical, anatomical, and biological factors
		**ORFAN study, Chan et al., 2024 [74]**	Prospective study of >40,000 patients followed up for MACE and prognostic value of FAI evaluated	AI-Risk incorporates FAI Score to provide clinically meaningful risk classification for patients undergoing CCTA	QRISK3 was a better-than-expected predictor of cardiac mortality or MACE; lack of inflammatory biomarkers
		**Henry et al., 2025 [75]**	Prospective study of patients undergoing CCTA had clinical management decisions recorded before and after FAI and AI-Risk scores revealed	AI-Risk analysis led to reclassification and altered 33% of patient’s clinical management determined by QRISK3 and SCORE	Relatively small single-centre study; population at low risk; no outcome data

**Table 3:** Key studies applying artificial intelligence (AI) to nuclear cardiology imaging, organised by modality and clinical task: (1) prognosis and management decision support; (2) obstructive CAD detection; (3) quantitative PET perfusion and phenotyping; (4) CT-free attenuation correction; (5) inflammatory cardiomyopathies; (6) image-quality optimisation; and (7) cardiac amyloidosis detection. Columns summarise the study focus, citation, brief study design/approach, key outcomes, and main limitations as reported by the authors. Abbreviations: AI, artificial intelligence; AL, immunoglobulin light-chain (amyloidosis); ATTR, transthyretin (amyloidosis); CAC, coronary artery calcium; CAD, coronary artery disease; CT, computerised tomography; DL, deep learning; FDG, fluorodeoxyglucose; FFR, fractional flow reserve; ICA, invasive coronary angiography; ML, machine learning; MBF, myocardial blood flow; MFR, myocardial flow reserve; MPI, myocardial perfusion imaging; MPS, myocardial perfusion scintigraphy; PET, positron emission tomography; SPECT, single-photon emission computerised tomography; SPECT/CT, hybrid SPECT with computerised tomography; 99mTc, technetium-99m; TPD, total perfusion deficit.

Section	Condition/Focus	Study (ref)	Study Summary	Key Performance/Outcomes	Main Limitations
**Prognosis and Management Decision Support (SPECT MPI)**					
	Risk prediction	**Singh et al., 2022 [87]**	Prognostic explainable DL applied to SPECT MPI; large multi-site cohorts with external validation	Added incremental prognostic value beyond traditional quantitative approaches	Retrospective design; no CAC information, all-cause mortality assessed
	Stress-only SPECT imaging	**Hu et al., 2020 [88]**	ML model trained on >20,000 patients predicted safe cancellation of rest scans after stress SPECT	Higher prognostic safety than current clinical approaches to rest SPECT cancellation	Physician diagnosis used additional information; prospective clinical validation needed
	Revascularisation prediction	**Arsanjani et al., 2015 [90]**	Retrospectively trained ML combining clinical and quantitative SPECT features predicts revascularisation	Predicted early revascularisation with accuracy comparable to expert readers; better than standalone perfusion measures	Limitations of revascularisation; MPS protocol used high radiation; multicentre validation needed
	Reader decision support	**Miller et al., 2022 [85]**	Prospective study with readers interpreting images with and without DL decision support	Significantly improved diagnostic accuracy of MPI with DL implementation	Did not measure changes in reader confidence; variability in equipment
**Obstructive CAD Detection and Interpretation (SPECT MPI)**					
	Obstructive CAD detection	**Betancur et al., 2018 [83]**	DL trained on large multicentre SPECT polar maps to detect obstructive CAD	Outperformed conventional quantitative metrics (TPD) for per-patient and per-vessel CAD detection	Visual interpretation of stenosis; polar maps only from stress static images; retrospective datasets
	Upright + supine integration	**Betancur et al., 2019 [84]**	DL model combining upright and supine SPECT polar maps from large, multicentre datasets	Improved CAD detection compared with TPD; overcame single-view limitations	Visual stenosis ICA; lack of FFR measurements; limited prospective validation
	AI-enhanced perfusion scoring	**Miller et al., 2025 [86]**	DL outputs translated back into quantitative perfusion scores	Greater predictive ability for obstructive CAD than traditional analysis or DL alone	Requires further external validation; lack of FFR evaluations
**Quantitative PET Perfusion and Phenotyping**					
	MFR & phenotyping	**Yeung et al., 2022 [93]**	DL trained on retrospective PET polar maps to detect impaired MFR and classify risk traits	Identified impaired MFR and cardiovascular risk phenotypes	Observational design; limited interpretability of DL algorithm
**CT-free Attenuation Correction (SPECT)**					
	Attenuation correction without CT	**Shanbhag et al., 2025 [99]**	DL developed on multicentre cohort of >4800 patients; generating synthetic attenuation-corrected SPECT	Improved diagnostic performance for obstructive CAD	Retrospective study design
	Attenuation correction without CT	**Yang et al., 2025 [100]**	DL-based attenuation correction for SPECT developed retrospectively on >160 patients	Enhanced CT-free attenuation correction with the implementation of CT features	Preliminary testing only; single-centre study with single scanner; limited dataset
**Inflammatory Cardiomyopathies (FDG PET)**					
	Cardiac sarcoidosis	**Poitrasson-Rivière et al., 2024 [94]**	DL myocardial segmentation trained on 316 FDG PET studies	Improved clinical readability in >90% of cases; improved processing time	Retrospective study design on a small cohort
**Image Quality Optimisation: Dose Reduction, Denoising & Reconstruction (SPECT/PET)**					
	Dose reduction & denoising	**Aghakhan Olia et al., 2021 [95]**	DL denoising retrospectively applied to low-dose SPECT to predict standard projection data	Diagnostic quality preserved at half dose; 80% acceptability at quarter dose	Limited performance in higher-risk patients due to cohort
	Image denoising	**Sun et al., 2023 [96]**	Retrospective DL denoising of SPECT images	Improved image quality at reduced dose	Small cohort; lack of diagnostic information
	Multi-frequency denoising	**Du et al., 2024 [97]**	Retrospective DL denoising of 50 stress SPECT/CT scans	Multi-frequency denoising outperformed conventional methods	Limited clinical efficacy evidence
	PET denoising and positron range correction	**Xie et al., 2025 [98]**	Self-supervised DL trained on 9 healthy patients for denoising dynamic PET frames and positron range correction	DL could simultaneously denoise images and correct positron range	Only preliminary validation; lack of clinical validation
**Cardiac Amyloidosis Detection and Quantification (SPECT/SPECT-CT)**					
	Cardiac amyloidosis detection	**Miller et al., 2024 [101]**	Retrospective study of 299 patients (28% ATTR CA); DL developed for volumetric quantification of ^99m^Tc-pyrophosphate uptake	Diagnostic accuracy was excellent without manual intervention; clinical outcomes established	Only one radiotracer and 3 h images used; lack of clinical follow-up data
	Cardiac amyloidosis detection	**Mo et al., 2025 [103]**	Retrospective study of 290 multicentre patients with suspected CA; ML integrating SPECT/CT radiomics for CA diagnosis	Outperformed traditional metrics; accurately classified ATTR vs. AL subtypes	Selection bias due to retrospective nature; small cohort

**Table 4:** Studies evaluating artificial intelligence (AI) approaches in cardiac magnetic resonance (CMR) imaging, organised by application area: (1) automated cine analysis and reporting, (2) disease detection/phenotyping, (3) synthetic contrast for gadolinium-free scar assessment (Virtual Native Enhancement), (4) scar quantification and risk stratification (automated infarct/scar and microvascular obstruction mapping), (5) acquisition and reconstruction, and (6) tissue characterisation and perfusion. Columns summarise the study focus, citation, brief study design/approach, key outcomes, and main limitations as reported by the authors. Abbreviations: AI, artificial intelligence; ARVC, arrhythmogenic right ventricular cardiomyopathy; AUC, area under the receiver operating characteristic curve; CAD, coronary artery disease; CMR, cardiac magnetic resonance (cardiac MRI); CNN, convolutional neural network; cine, cine (dynamic) imaging sequence; DCM, dilated cardiomyopathy; DL, deep learning; EF, ejection fraction; HCM, hypertrophic cardiomyopathy; ICM, ischaemic cardiomyopathy; ICD, implantable cardioverter-defibrillator; LGE, late gadolinium enhancement; LV, left ventricle/left ventricular; MBF, myocardial blood flow; MI, myocardial infarction; ML, machine learning; MVO, microvascular obstruction; n, sample size; QA, quality assurance; RV, right ventricle/right ventricular; SCD, sudden cardiac death; T1, longitudinal (spin–lattice) relaxation time; UK Biobank, United Kingdom Biobank; VNE, Virtual Native Enhancement (deep learning–generated, LGE-like images without gadolinium contrast).

Section	Condition/Focus	Study (ref)	Study Summary	Key Outcomes	Key Limitations
**Automated Cine Analysis and Reporting**					
	Automated LV/RV function from cine CMR	**Bai et al., 2018 [106]**	Large retrospective cohort (n = 4875; UK Biobank) using fully convolutional networks for automated LV/RV segmentation across the cardiac cycle.	Small mean differences vs. manual volumes; Dice up to 0.94; LV/RV volumes, mass and EF obtained in seconds.	Trained on research, single homogeneous dataset; performance in rare disease, small datasets and across vendors needs further validation.
**Disease Detection—Cardiac Amyloidosis**					
	Cardiac amyloidosis diagnosis from LGE	**Martini et al., 2020 [108]**	Retrospective multi-view LGE CMR study using CNNs and handcrafted-feature ML to detect cardiac amyloidosis.	AUC ≈ 0.98; DL performance comparable to feature-based ML and expert reading.	Retrospective, single-modality; needs multicentre, multi-vendor validation and workflow impact assessment.
	Amyloidosis diagnosis by LGE radiomics	**Zhou et al., 2022 [118]**	Retrospective multicohort LGE CMR study applying radiomics features and ML to diagnose cardiac amyloidosis.	Radiomics-derived models accurately diagnosed cardiac amyloidosis across cohorts.	Retrospective; acquisition heterogeneity may limit generalisability; needs prospective, vendor-agnostic validation.
	Amyloidosis prognosis by LGE radiomics	**Zhou et al., 2024 [119]**	Multicentre retrospective LGE CMR study using radiomics features to predict all-cause mortality in cardiac amyloidosis.	Radiomics-based models better predicted mortality, adding prognostic information.	Cut-offs and feature sets not standardised; external and temporal validation required before routine use.
**Disease Detection—ARVC**					
	ARVC: CMR Task Force Criteria	**Bourfiss et al., 2023 [109]**	Retrospective ARVC cohort using ML on cine-derived RV metrics to approximate CMR Task Force Criteria.	Feasible automatic classification of Task Force Criteria from quantitative RV features.	Development/validation cohort; performance in borderline or screening populations not yet defined.
**Disease Detection—ICM vs. DCM**					
	ICM vs. DCM differentiation (cine radiomics)	**Deng et al., 2024 [111]**	Retrospective cine CMR radiomics and ML study to differentiate ischaemic from dilated cardiomyopathy.	Radiomics + ML differentiated ICM vs. DCM, addressing a common diagnostic challenge.	Pipelines require standardised acquisition; multicentre external validation still needed.
	Non-contrast cine radiomics: ICM vs. DCM	**Lasode et al., 2025 [120]**	Retrospective non-contrast cine CMR radiomics study separating ICM and DCM and detecting infarct-related scar.	Non-contrast cine radiomics differentiated ICM vs. DCM and identified infarct scar without gadolinium.	Requires harmonised protocols and prospective testing; generalisability across centres and scanners uncertain.
**Disease Detection—HCM**					
	HCM fibrosis prediction without contrast	**Pu et al., 2023 [114]**	Retrospective HCM cohort using cine radiomics to predict presence of LGE-detectable fibrosis.	Cine-only radiomics identified patients likely to have LGE fibrosis, potentially reducing contrast use.	Adjunct to, not replacement for, LGE; needs prospective multicentre validation.
	HCM scar screening (cine + DL)	**Fahmy et al., 2022 [115]**	Multicentre HCM study comparing radiomics vs. DL vs. DL-radiomics in detecting myocardial scar as a marker of HCM.	DL-radiomics combined outperformed other AI systems.	Clinical thresholds and integration into care pathways remain to be defined.
	HCM: SCD risk from LGE radiomics	**Wang et al., 2021 [116]**	HCM cohort with LGE CMR using radiomics of scar texture and shape to predict sudden cardiac death.	LGE radiomics predicted SCD and added prognostic value beyond conventional markers.	Feature sets and thresholds not standardised; needs validation within guideline-based risk algorithms.
	HCM: prognostic value of scar heterogeneity	**Fahmy et al., 2024 [117]**	Multicentre HCM study using LGE radiomics to quantify scar heterogeneity for outcome prediction.	Scar heterogeneity carried prognostic value and complemented traditional fibrosis metrics.	Requires harmonised radiomics pipelines and prospective demonstration of added value for ICD decisions.
**Synthetic Contrast (VNE)**					
	Gadolinium-free scar imaging in HCM	**Zhang et al., 2021 [121]**	HCM cohort combining native T1 and cine CMR with a DL model to synthesise LGE-like Virtual Native Enhancement images.	VNE produced scar images comparable or superior to LGE, enabling contrast-free scar assessment in HCM.	Focused on HCM; broader cardiomyopathy and multi-vendor validation still ongoing.
	VNE for infarct scar post-MI	**Zhang et al., 2022 [122]**	Post-MI cohort using DL-based VNE for contrast-free infarct size assessment.	VNE images correlated strongly with LGE for infarct size and showed better image quality.	Limited to ischaemic scar cohorts; long-term reproducibility and multicentre performance still under study.
**Scar Quantification and Risk Stratification**					
	Automated scar quantification in HCM	**Navidi et al., 2023 [124]**	Retrospective HCM cohort with LGE using an interpretable CNN for LV contouring and scar segmentation.	Automated scar percentage strongly correlated with expert analysis (r = 0.92).	Developed in HCM only; impact on risk stratification and ICD decisions not yet proven.
	Automated MI scar and MVO segmentation	**Schwab et al., 2025 [125]**	Retrospective MI CMR cohort using a DL pipeline for fully automated infarct and microvascular obstruction segmentation.	Generated automated infarct and MVO maps supporting post-MI risk assessment.	Requires prospective validation, robustness testing and workflow integration.
**Acquisition and Reconstruction**					
	Free-breathing DL cine vs. breath-hold	**Klemenz et al., 2025 [126]**	Clinical comparison of free-breathing DL-based real-time cine reconstruction vs. standard breath-hold cine.	Free-breathing DL cine achieved non-inferior image quality and LV volume accuracy, reducing breath-hold requirements.	Validated on specific sequences and scanners; tested on healthy volunteers only; performance in severe arrhythmia or extreme body habitus unclear.
	Fast single-shot cine with super-resolution	**Aziz-Safaie et al., 2025 [127]**	Clinical study using DL super-resolution reconstruction for highly undersampled single-shot cine CMR.	Enabled fast single-shot cine with preserved diagnostic quality and shorter examinations.	Protocol- and site-specific; requires broader multicentre and multi-vendor validation.
	Free-breathing accelerated CMR in young patients	**Zucker et al., 2021 [128]**	Paediatric and young adult cohort undergoing DL-accelerated free-breathing cardiac MRI.	Accelerated free-breathing scans provided diagnostic quality comparable to conventional methods.	Focused on young patients; generalisability to older, multimorbid populations and all vendors not established.
	Super-resolution 4D flow MRI	**Ferdian et al., 2020 [131]**	Methodological study (4DFlowNet) applying DL super-resolution and denoising to 4D flow CMR.	Enabled shorter 4D flow acquisitions while preserving key haemodynamic metrics.	Research-level; requires validation against invasive standards and across scanners before clinical use.
**Tissue characterisation and perfusion**					
	Accelerated T1 mapping (MyoMapNet)	**Guo et al., 2022 [134]**	Clinical validation of DL-based MyoMapNet for inline T1 estimation in four heartbeats.	Four-heartbeat T1 mapping showed accuracy comparable to conventional mapping with shorter breath-holds.	Requires dedicated mapping capability and software; cross-vendor validation and QA still needed.
	Inline quantitative perfusion mapping (development)	**Xue et al., 2016 [135]**	Methodological/early clinical work on inline pixel-wise myocardial perfusion (MBF) mapping at the scanner.	Demonstrated feasibility of fully automated inline quantitative perfusion mapping.	Early development; implementation and MBF standardisation depend on vendor support.
	Repeatability of automated MBF in healthy subjects	**Brown et al., 2018 [136]**	Healthy volunteer study assessing repeatability of fully automated inline MBF CMR.	Showed good repeatability of automated MBF measurements.	Limited to healthy subjects; extension to disease populations and centres required.
	Repeatability of automated MBF in suspected CAD	**Elshibly et al., 2025 [137]**	Patients with suspected CAD undergoing fully automated inline quantitative perfusion CMR.	Demonstrated repeatability of MBF in a real-world CAD cohort.	Outcome-based validation and cost-effectiveness still needed; dependent on software availability.
	Inline perfusion mapping in HCM	**Camaioni et al., 2020 [138]**	HCM cohort studied with inline perfusion mapping to assess microvascular dysfunction.	Revealed microvascular dysfunction and provided mechanistic insight into HCM.	Primarily used in specialised centres; thresholds and treatment implications continue to evolve.

## Data Availability

No new data was generated as part of this work.

## List of Abbreviations

99mTc	technetium-99m
AC	attenuation correction
AI	artificial intelligence
AL	immunoglobulin light-chain (amyloidosis)
ARVC	arrhythmogenic right ventricular cardiomyopathy
AS	aortic stenosis
ASCVD	atherosclerotic cardiovascular disease
ASiR-V	Adaptive Statistical Iterative Reconstruction–V (iterative reconstruction algorithm)
ASNC	American Society of Nuclear Cardiology
ATTR	transthyretin (amyloidosis)
AUC	area under the receiver operating characteristic curve
AUROC	area under the receiver operating characteristic curve
AVR	aortic valve replacement
CA	cardiac amyloidosis
CAC	coronary artery calcium
CAD	coronary artery disease
CCTA	coronary computed tomography angiography
CE	Conformité Européenne (CE marking)
CLAIM	Checklist for Artificial Intelligence in Medical Imaging
CMR	cardiovascular magnetic resonance (cardiac MRI)
CNN	convolutional neural network
CT	computed tomography
CT-FFR	computed tomography–derived fractional flow reserve
CTCA	coronary computed tomography angiography (alternative abbreviation used in draft)
CV	cardiovascular
DCM	dilated cardiomyopathy
DECIDE-AI	reporting guideline for early-stage clinical evaluation of AI decision-support systems
DL	deep learning
DLR	deep learning reconstruction
EANM	European Association of Nuclear Medicine
EAT	epicardial adipose tissue
ECG	electrocardiogram
EF	ejection fraction
EHR	electronic health record
ESC	European Society of Cardiology
EU	European Union
FAI	fat attenuation index
FBP	filtered back projection
FDA	Food and Drug Administration (United States)
FDG	fluorodeoxyglucose
FFR	fractional flow reserve
GAN	generative adversarial network
H2FPEF	diagnostic score for HFpEF (Heavy, Hypertensive, Atrial fibrillation, Pulmonary hypertension, Elder, Filling pressure)
HCM	hypertrophic cardiomyopathy
HFA	Heart Failure Association
HFA-PEFF	Heart Failure Association diagnostic algorithm for HFpEF (Pre-test assessment, Echo & natriuretic Peptide score, Functional testing, Final aetiology)
HHD	hypertensive heart disease
IBSI	Image Biomarker Standardisation Initiative
ICA	invasive coronary angiography
ICC	intraclass correlation coefficient
ICD	implantable cardioverter-defibrillator
ICM	ischaemic cardiomyopathy
IR	iterative reconstruction
IVUS	intravascular ultrasound
LAVI	left atrial volume index
LGE	late gadolinium enhancement
LSTM	long short-term memory (network)
LV	left ventricle/left ventricular
LVEDV	left ventricular end-diastolic volume
LVEF	left ventricular ejection fraction
LVESV	left ventricular end-systolic volume
LVH	left ventricular hypertrophy
MACE	major adverse cardiovascular events
MBF	myocardial blood flow
MFR	myocardial flow reserve
MI	myocardial infarction
ML	machine learning
MPI	myocardial perfusion imaging
MPS	myocardial perfusion scintigraphy
MRI	magnetic resonance imaging
MVO	microvascular obstruction
NHS	National Health Service (UK)
NIHR	National Institute for Health and Care Research (UK)
PACS	Picture Archiving and Communication System
POCUS	point-of-care ultrasound
QA	quality assurance
QC	quality control
QRISK3	cardiovascular risk score (version 3)
RCT	randomised controlled trial
RIS	Radiology Information System
RMWA	regional wall motion abnormality
RV	right ventricle/right ventricular
RVEDV	right ventricular end-diastolic volume
SCD	sudden cardiac death
SCORE	Systematic COronary Risk Evaluation risk score
SCOT-HEART	Scottish Computed Tomography of the HEART trial
SGLT2	sodium–glucose cotransporter 2 (inhibitor)
SPECT	single-photon emission computed tomography
SPECT/CT	single-photon emission computed tomography with computed tomography
T1	longitudinal (spin–lattice) relaxation time
Tc-99m	technetium-99m
TPD	total perfusion deficit
TRIPOD	Transparent Reporting of a multivariable prediction model for Individual Prognosis Or Diagnosis
TRIPOD-AI	TRIPOD extension for prediction models based on AI/machine learning
TTE	transthoracic echocardiography
UK	United Kingdom
US	United States
VNE	Virtual Native Enhancement (DL-generated LGE-like images without gadolinium)
